# Deep-learning-based high-resolution recognition of fractional-spatial-mode-encoded data for free-space optical communications

**DOI:** 10.1038/s41598-021-82239-8

**Published:** 2021-01-29

**Authors:** Youngbin Na, Do-Kyeong Ko

**Affiliations:** Department of Physics and Photon Science, Gwangju Institute of Science and Technology, Gwangju, 61005 Republic of Korea

**Keywords:** Fibre optics and optical communications, Optical physics

## Abstract

Structured light with spatial degrees of freedom (DoF) is considered a potential solution to address the unprecedented demand for data traffic, but there is a limit to effectively improving the communication capacity by its integer quantization. We propose a data transmission system using fractional mode encoding and deep-learning decoding. Spatial modes of Bessel-Gaussian beams separated by fractional intervals are employed to represent 8-bit symbols. Data encoded by switching phase holograms is efficiently decoded by a deep-learning classifier that only requires the intensity profile of transmitted modes. Our results show that the trained model can simultaneously recognize two independent DoF without any mode sorter and precisely detect small differences between fractional modes. Moreover, the proposed scheme successfully achieves image transmission despite its densely packed mode space. This research will present a new approach to realizing higher data rates for advanced optical communication systems.

## Introduction

Since the beginning of innovations in information technology, such as the Internet of Things, big data, cloud computing, and artificial intelligence, demand for high-capacity communication systems has been explosively growing. Despite significant improvements in the capacity by the use of wavelength- and polarization-division multiplexing techniques and the application of multilevel modulation formats^1–4^, exponentially growing data traffic is facing bandwidth crunch. Light orbital angular momentum (OAM), which is one of the spatial degrees of freedom (DoF), has been proposed as a potential solution to overcome the limitation^5,6^. Light beams with a helical wavefront originating from an azimuthal phase ${\text{exp}}\left( {im\varphi } \right)$ have an OAM of $m\hbar$ per photon, where $m$ is a topological charge (TC), $\varphi$ is the transverse azimuth angle, and $\hbar$ is the reduced Planck constant^7,8^. Due to the theoretically unlimited integer values of $m$, OAM modes potentially provide an infinite-dimensional space, either as data symbols representing M-ary numbers^9–13^ or as independent information carriers for multiplexing^14–19^. Indeed, many results have shown that it is possible to achieve data capacity of Tbit/s or up to Pbit/s level through OAM mode division multiplexing in conjunction with other multiplexing techniques, over both free space and fibers^14–16^. However, contrary to the expectation, OAM does not increase the total amount of information and does not outperform both conventional line-of-sight multi-input multi-output transmission and spatial mode multiplexing with a complete basis^10,20,21^. In fact, OAM is only a subset of the transverse laser spatial modes, and the number of available spatial modes is restricted by the space-bandwidth product of a given optical system^10,20,21^. Hence, effective solution is to use full spatial DoF, e.g., both radial and azimuthal modes of Laguerre-Gaussian beams^10,11^, and one could consider separating the modes into a fractional interval to increase the addressable mode number in the limited system.

Unlike physical quantities defined on a continuous parameter space, such as linear momentum, OAM eigenmodes form a discrete set of modes where $m$ takes only integer values due to the periodic nature of the angle variable^22^. However, there also exist modes with arbitrary fractional OAM, and it can be described by a coherent superposition of integer OAM modes^22–24^. In other words, despite the OAM range limited by the system space-bandwidth product, one can employ more OAM modes by reducing the OAM interval to a fraction. Various methods have been proposed for measuring and recognizing OAM modes separated by a fractional interval. Some representative ways are based on specifically designed phase elements, which range from a conventional fork hologram^25,26^ to an annular grating^27^, multifocal arrays^28^, and mode sorters utilizing optical coordinate transformation^29,30^. These methods can obtain quantitative values of fractional OAM by measuring either intensity or focal spot displacement, but there are drawbacks to be improved, such as a low resolution, precision, and a need for additional optical elements. Furthermore, these methods are severely affected by optical alignment. Lateral displacement of a demodulation element relative to the optical axis leads to the power leakage into the neighboring modes, altering the OAM spectrum^31^. These issues make it difficult to develop a reliable optical system for fractional OAM beams.

Recently, a convolutional neural network (CNN), the most preferred solution for image classification^32,33^, has drawn attention as an efficient tool for recognizing OAM modes and correcting phase distortion caused by atmospheric turbulence^34–39^. End-to-end recognition of the deep-learning allows the classification process to be performed with only the intensity profile of the target modes, which is the most distinctive feature compared to the existing methods that require additional components to extract phase information^35^. Translation invariance of CNN provides stable recognition performance regardless of lateral displacement of a detector to the optical axis, and even it is possible to impart several transform invariances, such as rotation and scaling, through preparation and augmentation of proper data samples^35,39–41^. Moreover, a recent study has experimentally demonstrated that fractional OAM modes can be precisely recognized with a resolution of 0.01^41^.

In this paper, we propose fractional modulation of laser spatial modes effectively to increase the available mode number in limited optical systems. In particular, a CNN decoder is applied to achieve high-resolution recognition of fractional modes. The generation and modulation of spatial modes carrying a data symbol are implemented by switching phase holograms displayed on a spatial light modulator (SLM). The transmitted laser modes are captured by a CCD camera and directly recognized in real time by the trained CNN. As a proof of concept, gray values are encoded in 256 spatial modes given by a combination of radial modes and OAM modes and then transmitted in the proposed optical link. Despite the extremely small differences between the fractional modes, our CNN decoder successfully recovers the transmitted data without any optical mode sorter like a fork hologram. Using the proposed deep-learning-based technique, we were able to achieve recognition accuracy higher than 99% for the test dataset and transmit images with correlations higher than 0.99 and error rates lower than 0.10%.

## Experimental setup and methods

### Experimental setup

**Figure 1:**
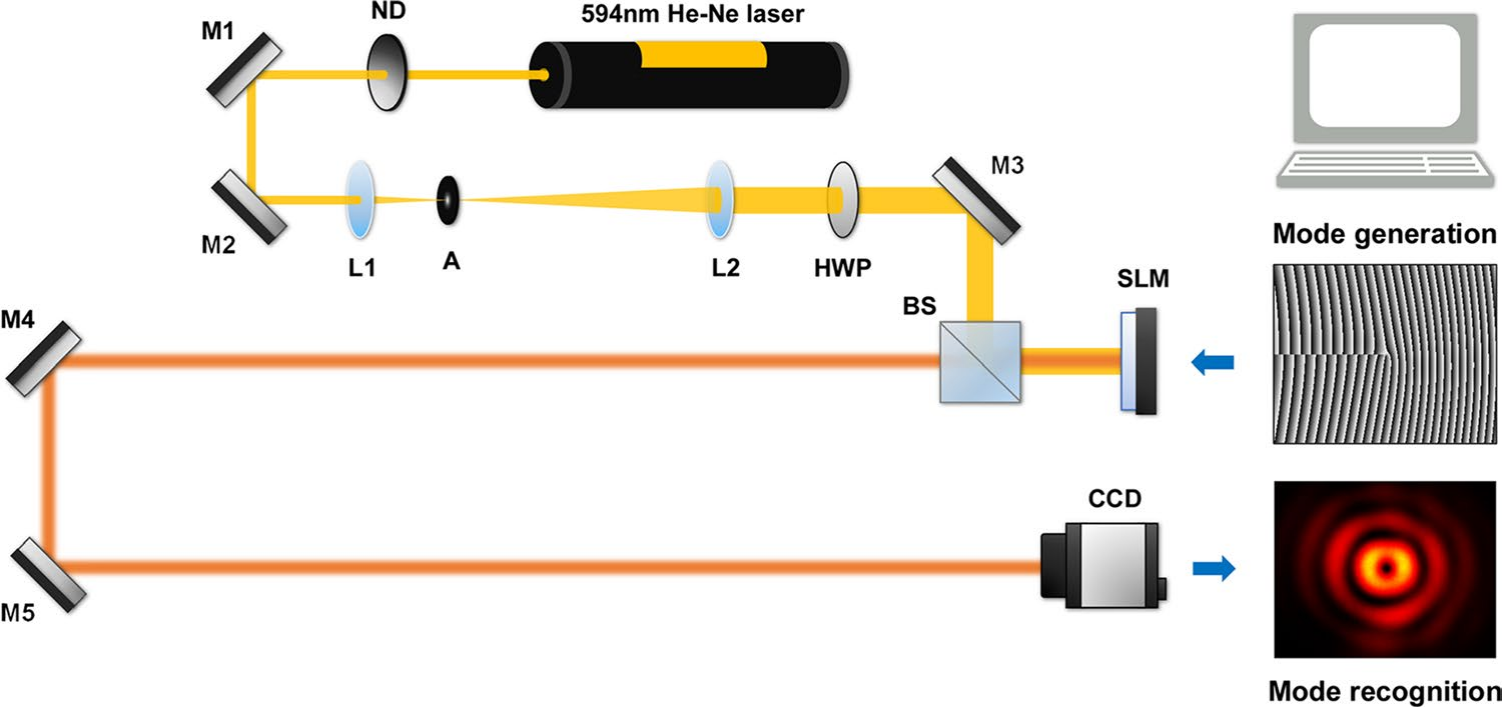
Top view of experimental setup: He–Ne laser of 594 nm wavelength; *ND* neutral density filter; *M* mirror; *L* lens; *A* aperture; *HWP* half wave plate; *BS* beam splitter cube; *CCD* charge coupled device. Propagation distance from the SLM to the CCD camera is 2.8 m. Each inset shows a hologram for mode generation and a captured intensity image for mode recognition.

Figure 1 schematically depicts the experimental setup to generate and recognize spatial modes of BG beams. A light beam emerging from a He–Ne laser of 594 nm wavelength is expanded and collimated by lenses L1 ($f = {\text{50 mm}}$) and L2 ($f = {\text{150 mm}}$). Then the laser beam of 2.1 mm radius is horizontally polarized through a half-wave plate and incident on an SLM. The SLM (PLUTO-VIS, Holoeye) is a reflective phase-only device with 1920 × 1080 pixels (8 μm pixel pitch). A spatial mode of BG beams is generated by the corresponding phase hologram displayed on the SLM. After 2.8 m propagation from the SLM, the generated BG beam is captured by a CCD camera of 768 × 576 pixels (8.3 μm pixel pitch). The captured intensity image is downsampled from 400 × 400 to 100 × 100 pixels. The down sampled image is fed to a neural network as an input, and the transmitted laser modes are predicted by the network in real time.

### Fractional modes of BG beams

Phase holograms for generating BG beams are given by^42^

1 $$ \begin{array}{*{20}c} {\Phi_{{k_{r} ,m}} \left( {r,\varphi } \right) = \exp \left[ {im\varphi + ik_{r} r} \right]} \\ \end{array} , $$

where $m$ is the azimuthal index that determines the order of the BG beam and a TC associated with light OAM. $k_{r}$ is the radial wavenumber that determines the spacing of the intensity rings and the non-diffracting distance given by^42^

2 $$ \begin{array}{*{20}c} {z_{\max } = w\frac{k}{{k_{r} }}, } \\ \end{array} $$

where $w$ is the radius of an input beam, and $k = 2\pi /\lambda$ is the propagation constant. Various BG modes can be generated by combining mode indices $m$ and $k_{r}$ in Eq. (1). Unlike the radial modes of which the mode index is a continuous parameter, a fractional OAM mode is given by a coherent superposition of all possible integer modes, not a single eigenmode, due to the discrete parameter $m$^22,24^. Spiral phase with a fractional index $l$ is expressed as^23^

3 $$ \begin{array}{*{20}c} {\exp \left( {il\varphi } \right) = \mathop \sum \limits_{m} c_{m} \exp \left( {im\varphi } \right) = \frac{{\exp \left( {il\pi } \right)\sin \left( {l\pi } \right)}}{\pi }\mathop \sum \limits_{m} \frac{{\exp \left[ {im\left( {\varphi + \phi } \right)} \right]}}{l - m}, } \\ \end{array} $$

where the complex coefficient $c_{m}$ of each integer mode is calculated by the orthonormal condition, and $\phi$ is a phase shift parameter that determines the orientation of the phase discontinuity and results in the rotation of the intensity profile; see Fig. 4b. Theoretically, the decomposition of fractional OAM modes includes all integer modes. However, the optical realization of the modes is restricted by the size of a physical aperture^24^ and above all, most of energy is distributed to adjacent 8 modes, e.g., $l = - 2$ to 5 in our case. In fact, the center ring structure of fractional beams, which provides crucial local features for each mode, consists of theses modes. The field distribution produced by the phase hologram of Eq. (1) can be described by the Fresnel diffraction integral^42^

4 $$ \begin{aligned} & u_{{k_{r} ,m}} \left( {\rho ,\theta ,z} \right) = \frac{1}{i\lambda z}\exp \left[ {ik\left( {z + \frac{{\rho^{2} }}{2z}} \right)} \right]\iint {u_{g} \left( {r,\varphi ,0} \right)\Phi_{{k_{r} ,m}} \left( {r,\varphi } \right)} \\ & \times \exp \left( {i\frac{{kr^{2} }}{2z}} \right)\exp \left[ { - i\frac{k\rho r}{z}\cos \left( {\varphi - \theta } \right)} \right]rdrd\varphi \\ & \approx \exp \left[ {im\left( {\theta - \frac{\pi }{2}} \right)} \right]\mathop \smallint \limits_{0}^{\infty } \exp \left[ { - \frac{{r^{2} }}{{w^{2} }} - ik_{r} + i\frac{{kr^{2} }}{2z}} \right]J_{m} \left( {\frac{k\rho }{z}r} \right)rdr, \\ \end{aligned} $$

where $z$ is the propagation distance, and $u_{{\text{g}}} = {\text{exp}}\left( { - r^{2} /w^{2} } \right)$ is the field distribution of an incident Gaussian beam. Here, we omitted the first term in the first line for the sake of simplicity and used the integral form of the high-order Bessel function^43^. Thus the resulting fractional BG beams with a radial index $k_{r}$ and an azimuthal index $l$ is given by

5 $$ \begin{array}{*{20}c} {U_{{k_{r} ,l}} \left( {\rho ,\theta ,z} \right) = \mathop \sum \limits_{m} c_{m} u_{{k_{r} ,m}} \left( {\rho ,\theta ,z} \right).} \\ \end{array} $$

**Figure 2:**
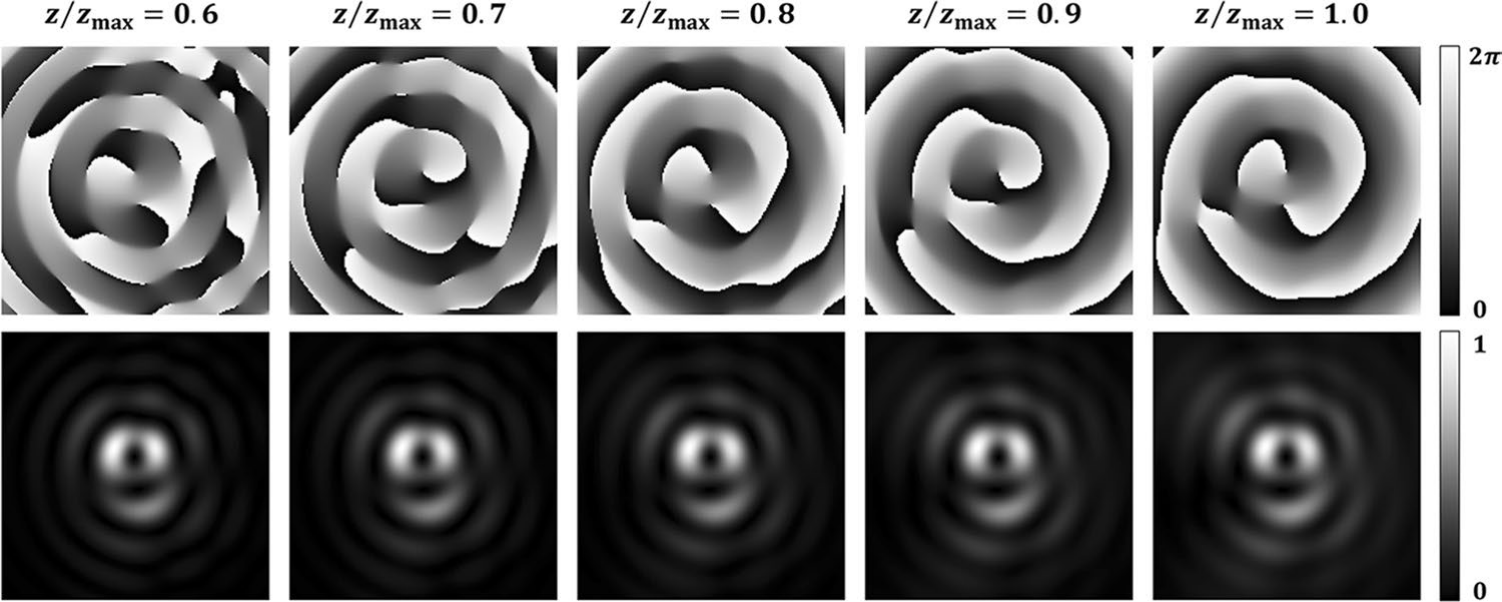
Numerically calculated phase and intensity profiles of a fractional BG beam at different positions. Propagation distance normalized to the non-diffracting distance is displayed on the top of the figure, and used parameters are as follows: $w = 2.1\;{\text{mm}}$, $\lambda = 594\;{\text{nm}}$, $k_{r} = 7.10$, and $l = 1.54$.

The Gouy phases of different OAM modes give rise to the unstable evolution of light beams emerging from a fractional spiral phase plate^24^. However, after a certain propagation distance, after $0.6z_{{{\text{max}}}}$ in our case, the transverse intensity profile of the mode does not change, as shown in Fig. 2. Besides, the fractional BG beam is diffraction-free during propagation over the finite distance, which indicates that the propagation properties of the ordinary BG beams are applied to Bessel beams carrying fractional OAM. Unlike integer OAM beams with a doughnut shape, distinctive structures of the fractional OAM modes provide various local features and allow CNN easily to discriminate differences between adjacent modes.

To demonstrate the feasibility of the densely encoded data transmission via a deep-learning method, we selected a total of 256 modes given by a combination of radial modes from $k_{r} = 7.10$ to $k_{r} = 7.80$ with $\Delta k_{r} = 0.10$ (8 modes) and OAM modes from $l = 1.03$ to $l = 1.96$ with $\Delta l = 0.03$ (32 modes). Meanwhile, a blazed phase grating and a surface correction hologram, made by a combination of Zernike polynomials, are additionally included in Eq. (1). The former separates the desired first diffraction order, and the latter compensates for the phase deformation caused by the spatial inhomogeneity of the SLM.

### Designed network structure and dataset preparation

**Figure 3:**
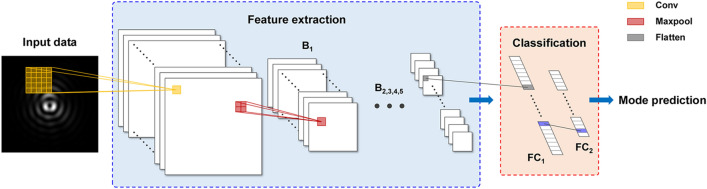
Structure of the designed CNN: B, block; FC, fully connected layer. A series of blocks which consist of a convolutional layer and a max pooling layer extract features describing the input object. The extracted features are classified through two FC layers, also referred to as dense layers. Finally, a spatial mode of the input is determined by a softmax activation function included in the last FC layer.

The designed CNN model comprises two parts for feature extraction and classification, as depicted in Fig. 3. The part of the feature extraction is constructed by 5 blocks, and each block consists of a convolutional layer and a max pooling layer. In this experiment, the number of blocks was determined considering the number of trainable parameters and model performance. The number of parameters in the first fully connected (FC) layer is proportional to the pixel size of input feature maps. Therefore, a model causes inefficiently large number of parameters if the size of feature maps is not scaled down properly. For this reason, 4 and 5 block models were taken into account, and the latter showed the lower validation loss despite similar training times. Multiple convolution kernels in the convolutional layer detect various local features of input images^32^. Then the extracted feature maps are processed by a nonlinear activation function. The purpose of the nonlinear activation function is to introduce non-linearity into the output, which allows the network to learn complex data and provide an accurate prediction^33^. Then the size of the activated feature maps is reduced by half through a max pooling layer, which allows the model to detect the weak variance coming from fractional mode separation^41^. The features extracted from the last block are connected to the classification part comprised of two fully connected (FC) layers. Finally, a softmax function included in the last FC layer yields the probabilities that the transmitted mode belongs to specific laser modes, and then a laser mode with the maximum value is determined to be a received mode; see Eq. (7).

Detailed information on the CNN is summarized in Table1, including the hyperparameters, output shape, and the number of trainable parameters. Each component in the third column of the table represents the number of convolution kernels, kernel size, activation functions, and zero paddings. For FC layers, each component represents the number of hidden units, also referred to as neurons of the layer, and activation functions. Additionally, He initialization useful for networks with the rectified linear unit (ReLU) as a nonlinear activation function was selected to initialize weight parameters^44^. The dropout layer placed in front of the first FC layer randomly sets 5% of its neurons to 0 for each training epoch to prevent overfitting^45^. It is activated only during the training time, which does not affect the inference.

**Table 1:** Summarized information on the designed CNN.

	Layer	Setting	Output shape	# of parameters
B1	Conv2D_1	64, (5, 5), ReLU, padding	(100, 100, 64)	1,664
	Maxpool2D_1	(2, 2)	(50, 50, 64)	0
B2	Conv2D_2	64, (4, 4), ReLU, padding	(50, 50, 64)	65,600
	Maxpool2D_2	(2, 2)	(25, 25, 64)	0
B3	Conv2D_3	128, (3, 3), ReLU, padding	(25, 25, 128)	73,856
	Maxpool2D_3	(2, 2)	(12, 12, 128)	0
B4	Conv2D_4	128, (3, 3), ReLU, padding	(12, 12, 128)	147,584
	Maxpool2D_4	(2, 2)	(6, 6, 256)	0
B5	Conv2D_5	256, (3, 3), ReLU, padding	(6, 6, 256)	295,168
	Maxpool2D_5	(2, 2)	(3, 3, 256)	0
–	Flatten	–	(2304)	0
–	Dropout	5%	(2304)	0
FC1	Dense_1	512, ReLU	(512)	1,180,160
FC2	Dense_2	256, softmax	(256)	131,328

To train and test the CNN, 51,200 intensity profiles with 200 images per mode are prepared. These 200 images have different phase shifts from 0.01 to 2.00π, respectively. Of the 200 images, 100 images, corresponding to phase shifts $\phi = 0.02,{ }0.04,{ } \ldots ,{ }2.00\pi$, are used for training the model, and the others for testing. Additionally, 20 images with different phase shifts from 0.05 to 1.95π per mode are prepared as a validation set. The role of the validation set is to provide an unbiased evaluation of the model during the training process. Meanwhile, the test set is used to assess the prediction accuracy of the final model. All images are resized from 400 × 400 to 100 × 100 pixels for computational efficiency and preprocessed for supervised learning.

## Experimental results and discussion

### Measurement of generated BG beams

**Figure 4:**
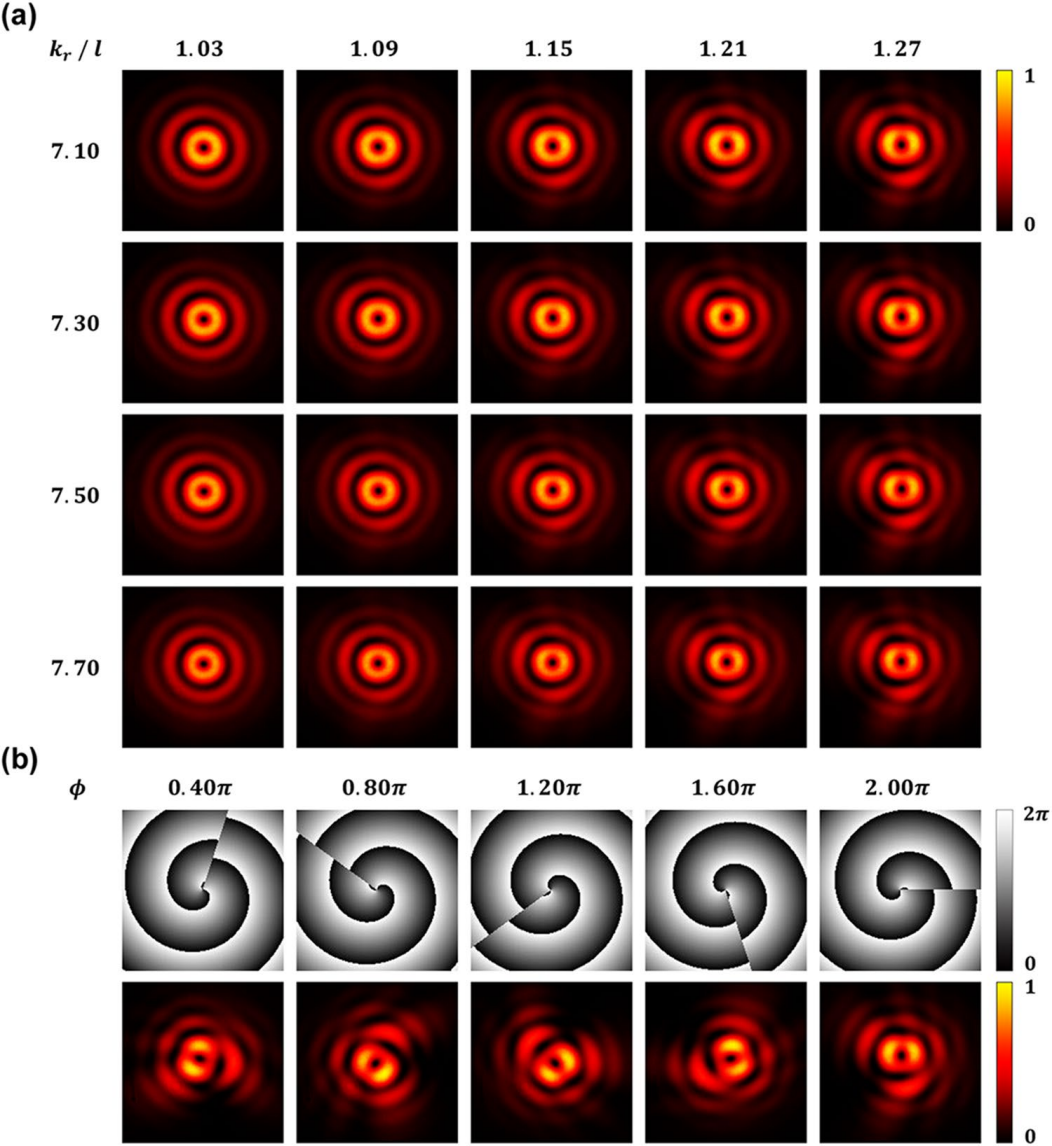
Experimental generation of fractional BG beams. (**a**) Some examples of the measured intensity profiles and (**b**) phase shift modulation of the fractional BG beam with $k_{r} = 7.10$ and $l = 1.45$.

Figure 4a displays captured intensity profiles of fractional BG beams generated with different mode indices. Here, pseudo-color, which varies smoothly from black to red and then yellow, is used for visual clarity, but the format of actual images is 8-bit grayscale. Only a few of all 256 spatial modes are presented to show and highlight the tiny variation caused by the fractional mode separation. As the radial wavenumber increases, the spacing between intensity rings is reduced. The intensity of the center ring decreases while the outer ring slightly brightens. Meanwhile, a spiral phase with a fractional TC results in exotic intensity distribution. As the charge increases from $l = 1$, the central ring of high-intensity forms two brighter light petals, and the second ring is transformed into a relatively dark and distorted four-petal structure. The angular position of those intensity peaks depends on the applied phase shift. The lower one of the four petals gradually approaches the central ring. Although not included in Fig. 4a, the petal moves further inward and then merges with the center ring. Finally, the intensity profile becomes a multiple ring structure again at $l = 2$. Using various convolution kernels, the deep-learning model detects invisible variations in local intensity distribution caused by the fractional mode separation. Then it gives the prediction, with high accuracy, based on the decision boundary formed by training data.

Figure 4b depicts the influence of the applied phase shift on the intensity distribution of fractional BG beams. As described above, the intensity profiles of the generated BG beams are rotated counterclockwise by the amount of the phase shift, which means that phase modulation applied to fractional modes is explicitly recognizable. In other words, unlike the canonical integer vortices with the rotational symmetry, broken symmetry of the fractional modes can provide an additional DoF for information coding. It will be a new kind of phase-shift keying technique that exploits phase-shifted fractional modes as M-ary symbols.

### Training of neural network and performance test

The CNN training and prediction are performed on the commercial GPU system (GPU: RTX 2060; CPU: i7-9750H) using Keras framework. The parameters of CNN are trained using the Adam optimizer with batch size 50 for 50 epochs. The Adam optimizer is a stochastic gradient descent method, which is based on adaptive estimation of moments^46^. It is computationally efficient, making it suitable for networks with large parameters. The weight parameters for the spatial mode recognition are updated to minimize a loss (objective) function during the training process. The loss function used for this purpose, i.e., for the multiclass classification problem, is the averaged categorical cross-entropy, which is given by^33,41^

6 $$ \begin{array}{*{20}c} {L = - \frac{1}{N}\mathop \sum \limits_{i = 1}^{N} \mathop \sum \limits_{j = 1}^{n} t_{j}^{\left( i \right)} \log \left( {S_{j}^{\left( i \right)} } \right), } \\ \end{array} $$

where $N$ is the total number of data to be considered, $n$ is the total number of classes, i.e., representing 256 spatial modes, and index $j$ represents each mode. For example, 1-32th classes correspond to modes with $k_{r} = 7.10$ and $l = 1.03$ to $l = 1.96$, and next 32 classes correspond to modes with $k_{r} = 7.20$ and $l = 1.03$ to $l = 1.96$. Meanwhile, $t_{j}^{\left( i \right)}$ is the *j*th element of a target label vector. The label vector is a vector of size *n* with one on the element corresponding to a specific spatial mode and zeros elsewhere. For example, in our experiment a label vector of the spatial mode with $k_{r} = 7.10$ and $l = 1.03$ is $\left\{ {1, 0, \ldots , 0} \right\}$, and that of the spatial mode with $k_{r} = 7.10$ and $l = 1.06$ is $\left\{ {0, 1, 0, \ldots , 0} \right\}$. $S_{j}^{\left( i \right)}$ is the output of a softmax activation function, which represents the probability that the *i*th sample belongs to the *j*th class, and it is given by^33^

7 $$ \begin{array}{*{20}c} {S_{j} = \frac{{\exp \left( {y_{j} } \right)}}{{\mathop \sum \nolimits_{m = 1}^{n} \exp \left( {y_{m} } \right)}}, } \\ \end{array} $$

where $y_{m}$ is the input of the *m*th unit, and $n$ is the total number of classes as described above. The initial learning rate is 0.001, which is reduced by half whenever validation loss does not be improved for 5 epochs. After the CNN training, prediction time was measured to be $t_{p} < 4 {\text{ms}}$ and it is shorter than the switching time of the SLM. In other words, our CNN model can decode all transmitted data without a delay of signal, which indicates that real-time prediction of laser spatial modes is possible. The calculation speed is affected by the Floating-point Operations Per Second (FLOPS) of the computing system^38^. Here, we employed RTX 2060 of a laptop system, and the FLOPS of a RTX 3080, one of the recent advanced GPU, is about four times higher. It implies that using a more advanced GPU can shorten the prediction time to sub-ms. In other words, the mode detection using the proposed model can be sufficiently applied to devices with fast switching speed, such as a digital micromirror device (DMD). The DMD is a pixelated device of micromechanical mirrors individually switched “on” or “off” states. The device is used as a binary amplitude mask for beam steering and shaping, and a fast refresh rate of up to a few kilohertz or faster is its typical characteristic^47^.

**Figure 5:**
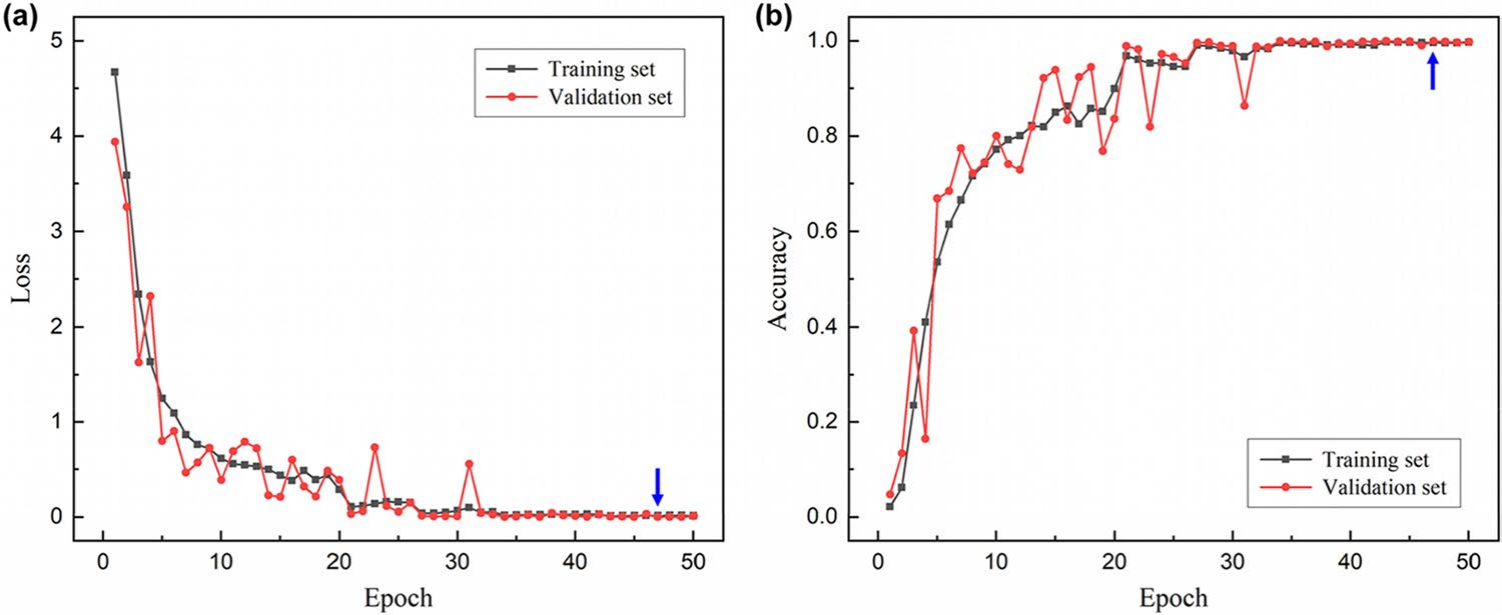
Training results of the designed CNN. (**a**) Loss and (**b**) accuracy curves for the training set and validation set. Blue arrows indicate the 47th epoch where the validation loss is minimum. Validation loss and validation accuracy were 6.46e−4 and 99.9% at the epoch.

Figure 5 shows the loss and accuracy curves for the training and validation sets as a function of the training epoch. Here the accuracy is defined as the number of correctly classified samples divided by the total number of samples. As shown in Fig. 5a, the model suitably learns the training data and performs well on new, unseen data without overfitting. Note that the validation set never participates in the training process and is used only to evaluate the model every epoch. Minimum validation loss 6.46e−4 was achieved at the 47th epoch, and the validation accuracy was 99.9%. The model and weight parameters that had achieved the minimum validation loss were saved and used for inference.

**Figure 6:**
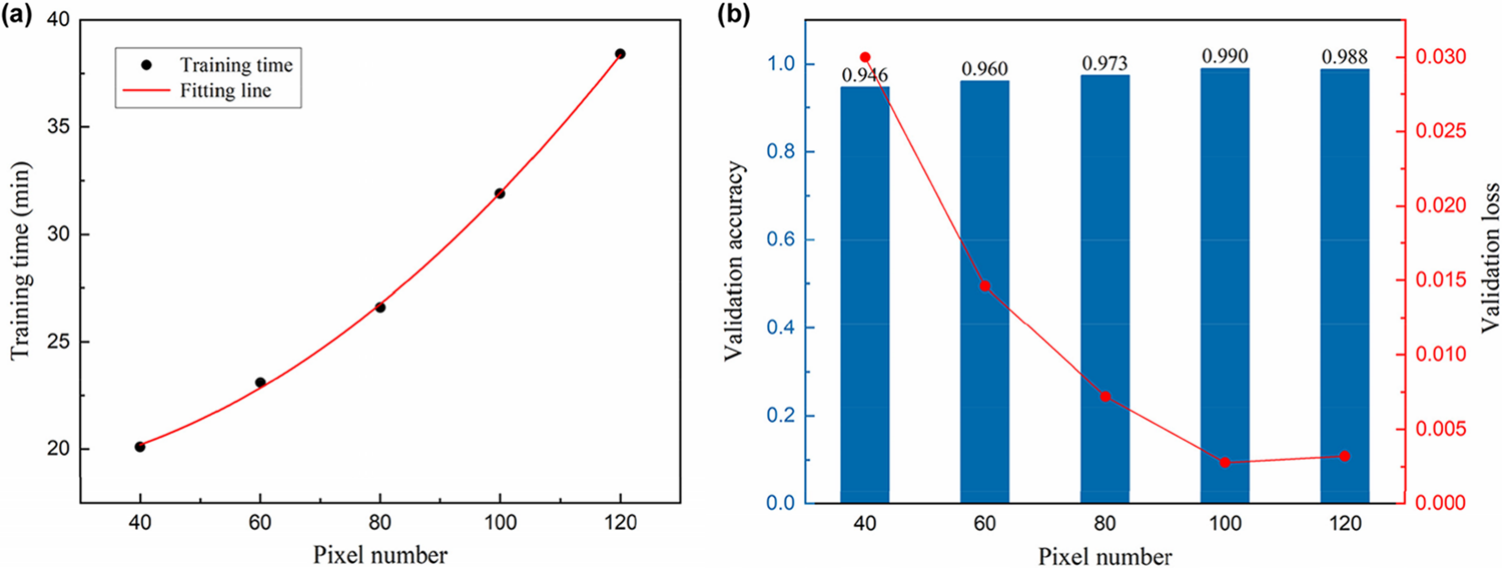
Performance evaluation of the number of input image pixels. (**a**) Time taken to train for 50 epochs and (**b**) validation loss and validation accuracy. All data show mean values of 10 training results per pixel number.

In addition to the recognition accuracy of the model, computational speed is a critical factor for a communication purpose and real-time prediction. For this reason, we investigated the influence of the input pixel size on the training time and model performance. The image set is resized from 400 × 400 to 5 different pixel numbers, from 40 × 40 to 120 × 120 pixels, using the open-source computer vision library (OpenCV) and fed to the network for training. As can be seen in Fig. 6a, the training time is proportional to the square of the pixel number, which is reasonable when considering that an image is a 2-d matrix. Figure 6b shows the evaluation results obtained with different pixel numbers. A minimum loss of 2.78e-3 and a maximum accuracy of 99.0% were achieved at pixel number 100. Because of a lack of information, the models trained with samples of a resolution lower than 100 × 100 pixels show poor performance, i.e., underfitting has occurred. On the other hand, the dataset of 120 × 120 pixels gives rise to the overfitting of the model, which means that the model has learned too many details like noise and may not be able to predict new data in the future. Regularization techniques such as l2 regularization, dropout, and batch normalization can mitigate the overfitting^45,48,49^, but using a dataset of 100 × 100 pixels is more efficient for model training and real-time prediction.

**Figure 7:**
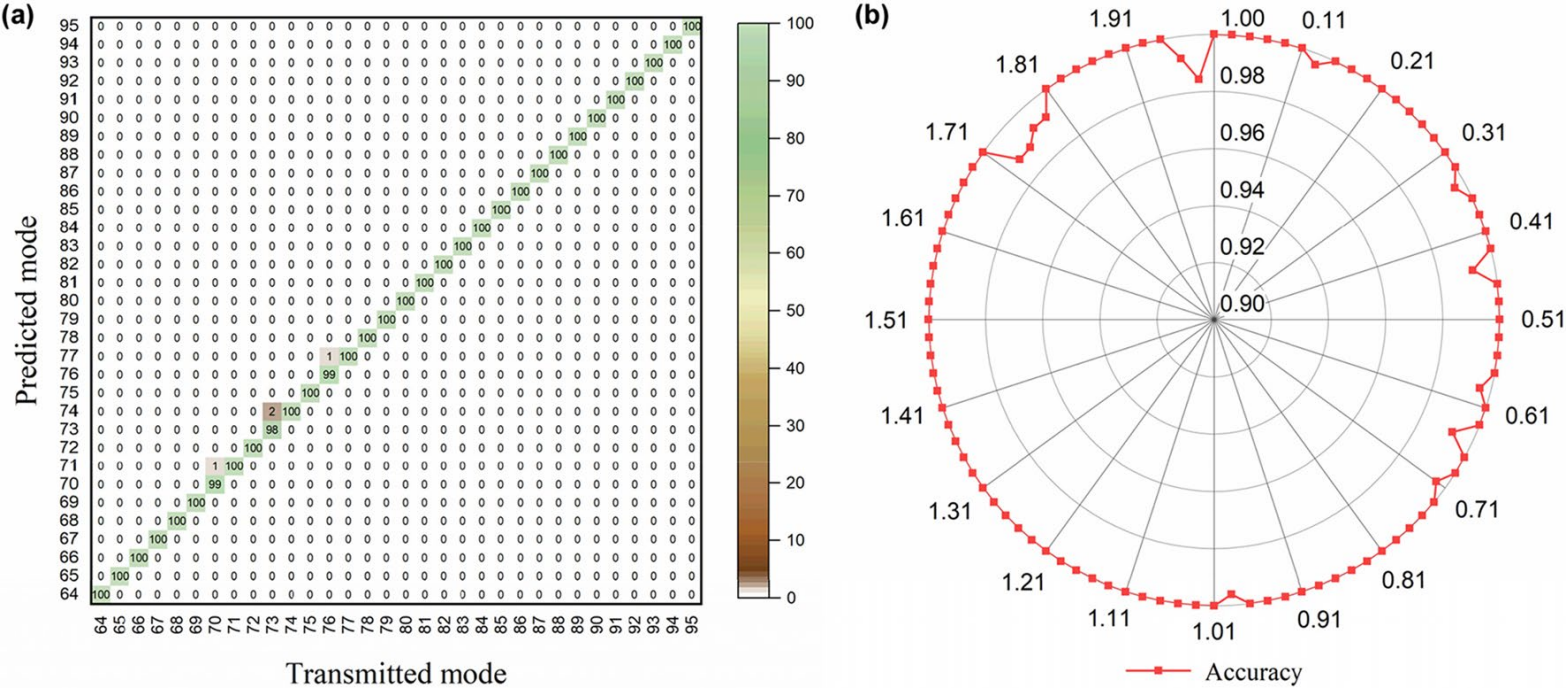
(**a**) A part of the confusion matrix obtained with a test set and (**b**) a graph of test accuracy versus the applied phase shift. Displayed numbers from 64 to 95 correspond to spatial modes given by combination of a radial wavenumber 7.30 and OAM modes from 1.03 to 1.96. The values inside the matrix represent the number of recognized spatial mode. The diagonal elements of the matrix indicate that the transmitted mode is correctly recognized. A unit of the phase shift here is rad/π.

With the trained model described above, an additional test was implemented to demonstrate the classification performance. Figure 7a shows a part of the confusion matrix obtained from the prepared test dataset. The numbers displayed on axes represent spatial modes given by a combination of a radial mode 7.30 and OAM modes from 1.03 to 1.96, which is employed for the sake of simplicity. The diagonal elements indicate that the transmitted mode is correctly classified. Only 25 of the 25,600 images make errors, which are because of the adjacent modes, and the measured test accuracy is 99.9%. The experimental results demonstrate that the trained model can simultaneously identify two independent spatial modes, without any optical mode sorters like a fork hologram. Besides, the model accurately recognizes extremely small differences between fractional modes regardless of the applied phase shift, as can be seen in Fig. 7b. It means that the OAM phase-shift keying proposed in subsection of measurement of generated BG beams could be combined to encode more data without degradation of the recognition accuracy. This is a distinct advantage compared to the optical coordinate transformation method, which shows different output results by the extrinsic OAM component of fractional OAM beams depending on the orientation angle of the phase discontinuity^30^.

### Image transmission by using spatial mode encoding

As a proof of concept, 8-bit grayscale images are transmitted, pixel by pixel, through free space. 8-bit encoding is performed with 256 spatial modes separated by fractional intervals, which demonstrates the possibility of super dense optical communication assisted by laser spatial modes and deep-learning. As shown in Table 2, 32 OAM modes with the same radial wavenumber are assigned to 32 levels of the 256 gray levels. For example, laser spatial modes $\left( {k_{r} = 7.10, \;l = 1.03} \right)$ and $\left( {k_{r} = 7.10,\; l = 1.06} \right)$ are assigned to 0 and 1, respectively. In other words, 256 different laser spatial modes act as 256-ary symbols.

**Table 2:** 8-Bit encoding scheme.

	0–31	32–63	64–95	96–127	128–159	160–191	192–223	224–255
$k_{r}$	7.10	7.20	7.30	7.40	7.50	7.60	7.70	7.80
$l$	1.03–1.96							

Image transmission is performed according to the following procedure. First, an image is transformed into a 1-d vector comprised of a series of 256-ary numbers, and the data encoding is done by switching the phase holograms corresponding to each value. At this time, start and stop frames specifying the beginning and end of each data stream are appended every 20 pixels, i.e., 20 symbols, to prevent timing errors between the transmitter and receiver. After free-space propagation, the transmitted laser beam is captured by a CCD camera placed at the receiver, and the trained CNN model decodes the data in real time from the received intensity image.

**Figure 8:**
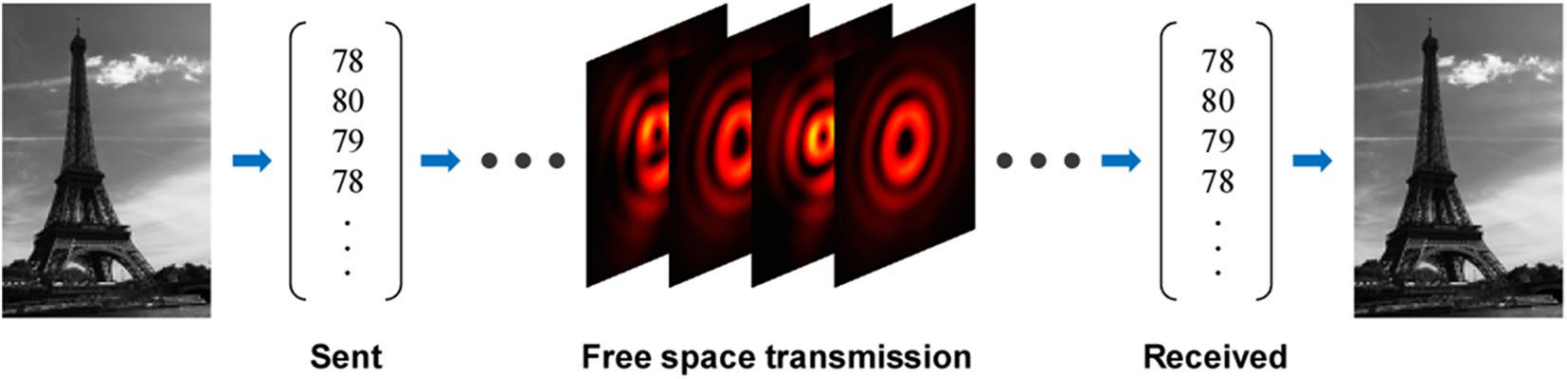
Image transmission in the proposed optical link. An Eiffel Tower gray image of 100 × 150 pixels is transmitted and received. An error rate defined as the ratio of the number of incorrect pixels to the total number of pixels was 0.05%.

Figure 8 shows the one result of image transmission performed using the proposed optical link. As a test sample, an Eiffel Tower image of 100 × 150 pixels was sent. Using the proposed 8-bit encoding scheme, we were able to process a total of 120,000 binary data into 15,000 encoding symbols. The measured error rate was 0.05%, and the errors occurred only at 8 pixels: two due to the nearest OAM mode, eight due to the nearest radial mode. A correlation coefficient between the transmitted image and received image was higher than 0.99, which implies that the reconstructed image is almost identical to the transmitted one. Despite the successful image reconstruction with the very high correlation coefficient, a wrong prediction caused by the adjacent radial mode is not negligible. However, one can reduce the error through multiple measurements or proper post-processing algorithms^50^. For example, a simple way is to conduct image transmission multiple times and extract either the most frequently occurring number or average value per each pixel. The image was transmitted four times consecutively to investigate not only long-term stability but the error reduction, and the error rate of the reconstructed image by extracting the most frequent number per each pixel was 0.

Despite the impressive performance of the deep-learning-based optical link, it is necessary to discuss some critical issues for future practical applications. One thing is the transmission speed associated with data encoding and decoding. Encoding speed is determined by the frame rate of an SLM, which limits the symbol rate of the communication link. In the experiment, the hologram switching rate is set to 2.5 Hz, transmitting 20 bits of information per second. Even if we use the maximum rate of the SLM (60 Hz), the highest achievable data rate is only 480 bits per second. However, the demand for high-speed OAM switching can be addressed by using tunable integrated OAM devices^51,52^ or DMD^53^, which can increase the modulation rate up to a few tens of kilohertz or faster. Meanwhile, a camera with a frame rate faster than the refresh rate of a used modulator is needed to capture and decode laser spatial mode varying over time, and it could be addressed by using a CMOS camera. Assuming that the frame rate of a system, made up of a DMD and a CMOS camera, is 1 kHz, the transmission speed that can be achieved with 256-ary spatial mode encoding is just 8 (1 × 8) Kbit/s. However, it is possible to increase the number of bits per symbol by employing more spatial modes and combining the OAM phase shift keying. Note that we achieved 8-bit encoding in an OAM subspace with the radial mode and deep-learning-based high-resolution recognition method. It indicates that the proposed link can transmit numerous data even if the size of the aperture restricts the range of available OAM modes. Besides, the capacity of the communication system can be increased further by combining other photonic DoF (wavelength and polarization)^10^. In other words, the speed can be doubled by combining two polarization states, and *N* times further by adding phase shift modulation of fractional modes. The maximum data speed is estimated to be a few hundred Kbit/s. Note that an optical link with a single wavelength was considered. This shows a relatively lower speed compared to fiber-optic communication systems, but the proposed OAM encoding scheme is able to improve physical layer security due to its inherent characteristic that does not depend on mathematical or quantum–mechanical encryption methods^5,54^. Therefore, the proposed optical link would also be applicable for military communication systems requiring high security.

For long-distance outdoor links, there exists atmospheric turbulence that distorts the structured phase and intensity and degrades the link performance. Methods of adaptive optics, based on a wavefront sensor^55^ or deep-learning^37,38^, can be taken into account to compensate for the deteriorating effects of turbulence. In the case of strong atmospheric turbulence, an active method based on ultrashort high-intensity laser filaments can produce a cleared optical channel by opto-mechanically expelling the droplets out of the beam area^56^. Besides, it is possible to achieve long-range propagation by means of nonlinear self-channeling of high-power laser pulses^57^. Compared to the ordinary spatial modes of an integer mode index, fractional modes are much sensitive to the external perturbation. We will investigate the transmission performance of the fractional mode encoding in different atmospheric turbulence levels and an effective method to mitigate intermodal crosstalk.

## Conclusion

In conclusion, we experimentally demonstrated that both radial and azimuthal modes of BG beams, with the fractional mode spacing, can be used to transmit information over free space. 8-bit data are densely encoded in 256 spatial modes and successfully decoded by a deep-learning classifier. To achieve this, we first trained the designed neural network based on Alexnet architecture and tested its performance. The recognition accuracy of different fractional modes was nearly 100%, which demonstrates that the deep-learning decoder can simultaneously identify two independent spatial modes and accurately recognize extremely small differences between adjacent modes. Furthermore, the translational and rotational invariance of the trained CNN provided a reliable, stable model performance despite the sensitivity of fractional OAM beams to the optical alignment. Then, we transmitted a 100 × 150 grayscale image, a total of 120,000 bits, encoded with 15,000 data symbols by switching phase holograms, and the transmitted data was successfully recovered in the proposed optical link. In addition to the fractional modulation, the explicit phase shift applied to fractional OAM beams could provide an additional degree of freedom for information coding, which is achievable without degrading the performance of the proposed method. There are challenges to be overcome, such as modulation rate and atmospheric turbulence, but the proposed fractional mode encoding/deep-learning decoding scheme will provide an effective way to meet the growing demand for data traffic.
